# Ropivacaine Plasma Concentrations after 192-Hour High Dose Epidural Ropivacaine Infusion in a Pediatric Patient without Side Effects

**DOI:** 10.1155/2018/9150980

**Published:** 2018-07-11

**Authors:** Glenn van de Vossenberg, Selina van der Wal, Andrea Müller, Edward Tan, Kris Vissers

**Affiliations:** ^1^Department of Anesthesiology, Pain and Palliative Medicine, Radboudumc, Nijmegen, Netherlands; ^2^Department of Surgery, Radboudumc, Netherlands

## Abstract

This case report discusses continuous epidural administration of ropivacaine 0.56 mg kg^−1^ h ^−1^ for 8 days in a 7-year-old trauma patient to prevent pain, after performing a lower right and upper left leg guillotine amputation. Venous sampling after 8 days revealed bound and unbound ropivacaine concentrations of 1.1 mg/l and 0.06 mg/l in plasma, respectively. Arterial sampling for bound and unbound ropivacaine was 1.2 mg/l and 0.05 mg/l in plasma, respectively. In this case report, long-term high dose epidural infiltration of ropivacaine did not result in severe side effects or complications. Further studies are needed to explore safety of these concentrations in larger populations of children.

## 1. Introduction

Since cocaine was introduced in the 19^th^ century by Carl Köller and Sigmund Freud, the use of local anesthetics (LA) has evolved enormously. Local anesthetics can be used to produce local, locoregional, and neuraxial nerve blockade. Binding of LA to various subtypes of sodium channels (Na_v_) in the nervous system produces nerve blockade. Currently 7 subtypes of Na_v_ are known in the nervous system. Blocking on these receptors can potentially cause minor side effects such as a metallic taste or tingling, but also severe side effects such as seizures and cardiac arrest. All these symptoms and adverse effects are referred to as local anesthetic systemic toxicity (LAST) [1]. In pediatrics little is known about the absolute maximum dosages for epidural infusion of ropivacaine. Only two studies [2, 3] have been performed in pediatrics assessing the save use of ropivacaine given via continuous epidural infusion for maximum dose of 0.4 mg kg^−1^ h^−1^. In this case report we present and discuss a case of continuous administration of ropivacaine 0.56 mg kg^−1^ h^−1^ in the epidural space for over 8 days to control severe pain after bilateral amputation and reveal its concentration in venous and arterial plasma after 8 days. Written informed consent was obtained from the patient's parent for publication of this report.

## 2. Clinical Presentation

A previously healthy 7-year-old male presented to a community hospital with severe lower extremity trauma due to accident with a truck. The patient was instantly brought to the OR and the surgeon performed a lower right leg and an upper left leg guillotine amputation. During surgery the patient received one litre of crystalloids, two units of packed cells, and one unit plasma and remained hemodynamically stable with low noradrenaline dosage. For postoperative analgesia a n. ischiadicus and a n. popliteal catheter were placed during surgery in the left and right lower extremity, respectively. Since much postoperative pain was expected ropivacaine infusion of 0.2 mg kg^−1^ h^−1^ was started over each catheters (0.4 mg kg^−1^ h^−1^ in total), postoperatively. Intravenous infusion of esketamin 0,2 mg kg^−1^ h ^−1^ and morphine 20 mcg kg^−1^ h^−1^ was started additionally. Initially peripheral catheters were preferred since less hemodynamic consequences were expected. Postoperative analgesia was generally sufficient scoring NRS 0 at rest and NRS 2 during movement. However, after one week his pain management was not sufficient anymore, most probably due to peripheral catheter manipulation after several debridements and stump closure on OR. Therefore, the peripheral catheters were removed and since the patient was hemodynamically stable and had no fever anymore a tunneled epidural catheter (L4-L5) was placed and ropivacaine 0.4 mg kg^−1^ h ^−1^ infusion was started epidurally. This resulted in adequate pain management during rest and additional morphine was stopped since it caused itching. However, during wound treatment the next day the patient experienced again nonacceptable pain. A bolus of esketamin did not reduce pain to acceptable levels. Moreover, infusion of esketamin is controversial since it may induce liver enzyme disorder and it was stopped [4]. One day after epidural placement a bolus of 10 ml ropivacaine 0.375% with 5 mcg of sufentanil resulted in adequate pain relief, and we increased continuous epidural infusion to 0,48 mg kg^−1^ h ^−1^ of ropivacaine. Initially a bilateral sensory block was present at T12/L1 without a motor block. We observed the patient in a medium care unit with continuously pulse oximetry, 3-lead ECG (no qualitative 12-lead ECG analysis) and blood pressure monitoring. Furthermore, our nurses are trained to recognize symptoms of LAST and the patient was monitored on a daily basis by the acute pain service. Since we did not observe symptoms of LAST whatsoever, no side effects of epidural bupivacaine 0.5 mg kg^−1^ h ^−1^ were described in literature [5], the department of pediatric anesthesiology approved these high concentrations, and adequate pain relief was obtained and these high dosages were accepted. After taking into account the weight loss due to amputation we were actually infusing at 0.56 mg kg^−1^ h ^−1^.

Since few studies are performed to assess intravenous ropivacaine concentration after subsequent epidural infusion for days in pediatrics, we obtained venous and arterial samples after 8 days of epidural ropivacaine infusion and continued epidural infusion. Venous sampling resulted in 1.1 mg/l and 0.06 mg/l bound and unbound ropivacaine concentration, respectively. Arterial sampling resulted in 1.2 mg/l and 0.05 mg/l bound and unbound ropivacaine concentration, respectively. To our knowledge there were no other laboratory tests which we could perform to monitor for ropivacaine toxicity, other than more frequent testing which we did not find ethical to do in a child.

After 11 days, our patient was transferred, with the epidural in situ, to another hospital closer to his hometown. In follow-up, we learned that the epidural catheter was luxated during transport and thus removed. At this moment, the pain was tolerated well. No long-term side effects such as paresthesia were observed.

## 3. Discussion

As far as we know, this case report is the first report on administering 0.56 mg kg^−1^ h ^−1^ ropivacaine epidurally for 8 consecutive days in a pediatric case with severe trauma and hence pain. Therefore, we sampled venous and arterial concentrations of ropivacaine after 8 days. The unbound and bound ropivacaine concentrations fractions showed an equal distribution in the arterial and venous compartment. However, Knudsen et al. [6] showed a 2-fold and 4-fold higher arterial than venous ropivacaine concentration after IV-infusion for bound and unbound fraction, respectively. Different concentrations of local anesthetics in the venous and arterial compartment were observed in other studies after extravascular administration as well and they show that an equilibrium was reached after 2h of extravascular administration [7, 8]. This effect is known as the so-called flip-flop effect and is caused by slow distribution of local anesthetics and is also identified in epidural infusion. This helps us understand why we found an equal distribution of plasma ropivacaine concentrations in the venous and arterial compartment in our patient.

Furthermore, Knudsen et al. showed first adverse effects for unbound ropivacaine at 0.15 mg/l in adults; the concentrations measured in our case were well below this concentration. The question what concentrations of ropivacaine are necessary to cause LAST symptoms in pediatrics cannot be directly answered with this comparison because of the different pharmacology between children and adults such as bigger volume of distribution in children (not important in this case since steady-state was attained) and a higher susceptibility for LA in peripheral blocks [9]. However, the higher concentrations measured in this case report are not causing LAST symptoms in this patient. Evidence is available that toxic concentration for bupivacaine is reached at 3.7 mg l^−1^ in children [10]. Unfortunately no study revealed thus far the toxic concentration of ropivacaine for children.

Concentrations we measured in this case report are in line with other studies by Bösenberg et al. [2] and Berde et al. [3]. Since they only assessed infusion for 24-72h, we showed what concentration of ropivacaine after 192h is not accumulating. This was also shown by Berde et al., who showed stable and decreased concentrations of unbound ropivacaine throughout epidural infusion. Because we only report one case one should be prudent with generalization, especially since we only sampled at day 8.

Gustorff et al. [11] described a case report where they performed ropivacaine continuous epidural infusion at 1.14 mg kg^−1^ h^−1^ with an optional bolus of 1.36 mg kg^−1^. After 70 hours they measured a total concentration of 1.54 ml l^−1^ in plasma. Unfortunately they did not mention the bound and unbound concentration, so it is difficult to compare our measurement with their data other than stating that their total concentration was higher than we found. This can be easily explained by the fact that they were infusing at higher rates and in addition had a bolus function.

Based on this case report we cannot advise to allow higher concentrations of epidural ropivacaine as advised in current literature in infants than 0.4 mg kg^−1^ h^−1^, since we only took one measurement and thus cannot describe the typical pharmacological profile of 0.56 mg kg^−1^ h ^−1^. Further studies are needed to explore safety of these concentrations in larger populations of children. However, we can conclude that we did not observe any side effect nor symptoms of LAST. Moreover, the unbound ropivacaine plasma concentrations we measured did not reach toxic levels if compared to current literature. So we advise clinicians to consider titrate ropivacaine infiltration to higher concentrations when pain treatment is difficult to manage. Of course, it is mandatory to monitor regularly for symptoms of LAST when higher concentrations of ropivacaine are infused epidurally. Moreover, clinical symptoms are most important to observe since it is known that large variability exists in serum free/total local anesthetic concentration and sensitivity to local anesthetics [7]. Unfortunately, only few laboratories offer the possibility of assessing the concentration of ropivacaine in plasma, so clinicians must take into account the fact that it might take some time (weeks) before ropivacaine concentrations are measured.
